# Farmer Burnout in Canada

**DOI:** 10.3390/ijerph16245074

**Published:** 2019-12-12

**Authors:** Andria Jones-Bitton, Briana Hagen, Stephen J. Fleming, Sandra Hoy

**Affiliations:** 1 Department of Population Medicine, University of Guelph, 50 Stone Road East, Guelph, ON N1G 2W1, Canada; bhagen@uoguelph.ca; 2 Department of Psychology, Faculty of Health, York University, 4700 Keele Street, Toronto, ON M3J 1P3, Canada; sfleming@yorku.ca; 3 School of Social Work, Laurentian University, 935 Ramsey Lake Road, Sudbury, ON P3E 2C6, Canada; shoy2@laurentian.ca

**Keywords:** agriculture, burnout, cynicism, exhaustion, farmers, professional efficacy

## Abstract

While farmers in several countries worldwide are reported to be at higher risk for poor mental health outcomes like chronic stress, depression, and anxiety, there is a paucity of research on burnout in farmers. This cross-sectional study used an online survey administered between September 2015 and February 2016 to investigate burnout (as measured by the Maslach Burnout Inventory–General Survey (MBI–GS)) amongst farmers in Canada. The specific objectives were to measure the three components of burnout (exhaustion, cynicism, and professional efficacy), and to explore potential associated risk factors, as well as to determine the prevalence of the different burnout profiles (engaged, ineffective, overextended, disengaged, and burnout). MBI–GS results were obtained from 1075 farmers. Approximately 70% of the study sample identified as male and 30% as female, and participants were from a variety of farming commodities. Scores for exhaustion, cynicism, and professional efficacy were all higher than international norms. While 43% of participants were classified as engaged, 44% were classified in the ineffective, overextended, or disengaged profiles (i.e., intermediate profiles on the engagement – burnout continuum), and 12% were classified in the burnout profile. Risk factor results highlighted the positive effects of farmer support from spouse/romantic partner, friends, and industry. Overall, the results from this study demonstrate cause for concern with respect to farmer burnout, suggest potential avenues for intervention, and serve as a call to action to better support farmers in Canada.

## 1. Introduction

Farmers in Canada, the United States, the United Kingdom, Australia, Finland, and Norway reportedly have increased risk of poor mental health outcomes, including high risk or levels of depression [1,2,3,4], anxiety [1,4], stress [4,5,6,7,8], psychological distress [4,9], and psychological morbidity [10]. Poor farmer mental health not only negatively affects the farmers themselves, but can also result in negative consequences for their families, farm productivity, and animal welfare [11,12,13]. Concerns around poor mental health of farmers are further heightened as an increased global emphasis is placed on the need for sustainable agriculture to meet the challenge of feeding 9 billion people by 2050 [14]. Aspects of sustainable agriculture must surely include the sustainability of the farmers themselves, and poor mental health in this population threatens sustainability.

Burnout is a particularly understudied area of farmer mental health. As described by Leiter and Maslach (2016), burnout “reflects a fundamental crisis in the psychological connections that people establish with work” (p. 91), and is a syndrome of exhaustion, cynicism, and low professional efficacy [15]. Burnout is associated with negative consequences to physical and psychological health, and has negative professional implications including job dissatisfaction, absenteeism, and presenteeism [16]. Hence, burnout in farmers not only poses personal risks to those affected, but could also have negative implications for farm productivity and business, and the on-going success of the agricultural sector. Published research on burnout in farmers is sparse, with reports only from France [17,18], Finland [8], and Switzerland [19]. 

The largest study of farmer burnout to date is that published by Reissig and colleagues, who investigated burnout in over 1300 farmers in Switzerland using the Cophenhagen Burnout Inventory [19]. Twelve percent of participants were characterized as having burnout. Numerous factors were reported to be associated with burnout, including interpersonal conflicts, financial stress, heavy workload, time pressure, lack of free time, and poor health [19]. A smaller study of 265 dairy farmers was conducted by Kallioniemi and colleagues in Finland [8]. The authors reported that 9% of participants had “severe burnout symptoms” and the most common stressors reported by participants were agricultural policy, the “treatment of farmers in society and media”, workload, unpredictability of farming, and animal disease [8]. Of the two studies in France, one involved qualitative interviews of 22 retired farmers [17], and the other investigated burnout amongst 104 dairy farmers using two of the three subscales of the Maslach Burnout Inventory–General Survey (MBI–GS) [18]. Unfortunately, the MBI–GS results were not reported in the latter study, although the authors reported negative professional and personal interactions as risk factors for burnout [18].

The Maslach Burnout Inventory (MBI) is recognized as the leading burnout measure [20] and the MBI–GS has been used in research with farmers in Finland [8] and France [18]. The MBI is grounded in a theoretical perspective that “views burnout as a psychological response to aspects of one’s daily experiences” [20] (p. 1). Unlike other versions of the MBI (e.g., the MBI–Human Services Survey), the MBI–GS “focuses on the performance of work in general regardless of the specific nature of that work” and it “defines burnout as a crisis in one’s relationship with work, and does not specifically focus on one’s relationships with people at work” [20] (p. 37). The MBI–GS measures burnout on three subscales: exhaustion, cynicism, and professional efficacy [20]; further details on the MBI-GS are described below. Recently, Leiter and Maslach used a latent profile analysis to characterize individuals to one of five profiles according to their MBI subscale score; these profiles are engaged, ineffective, overextended, disengaged, and burnout [15]. These profiles can be useful in detecting burnout earlier in its development and to provide more tailored intervention solutions for burnout [15].

The goal of the present study was to better understand burnout amongst farmers in Canada. The specific objectives were to: measure the three components of burnout (exhaustion, cynicism, and professional efficacy) and explore potential associated risk factors, as well as to determine the prevalence of the different burnout profiles (burnout, disengaged, overextended, ineffective, and engaged).

## 2. Materials and Methods

Between September 2015 and February 2016, a national cross-sectional study using an online questionnaire was conducted on farmer mental health in Canada; details on the study have been reported elsewhere [4]. Included in the questionnaire was the MBI–GS. The present study reports on the MBI data collected in this national survey.

### 2.1. Study Design

National and provincial agricultural organizations in Canada were contacted via email or telephone and asked to share the survey link with their members via emails, listservs, newsletters, and/or magazines. Social media, in particular Twitter, was also used to promote the survey. People were eligible to participate if they were a self-identified farmer in Canada (any agricultural community), able to read and write in English, and 18 years of age or older. At the participants’ choosing, they could provide their email address separate from their questionnaire responses to be included in a draw for one of three $250 cash prizes. The data were collected anonymously, and informed written consent was obtained at the start of the questionnaire. The study was conducted in accordance with the Declaration of Helsinki, and the protocol was approved by the University of Guelph Research Ethics Board (15-JN-007).

### 2.2. Questionnaire

The questionnaire included the MBI–GS, which is a 16-item self-report scale used to measure separately the three components of burnout: exhaustion (5 items), cynicism (5 items), and professional efficacy (6 items) [20]. Items are measured using a 7-point Likert scale (0–6). The exhaustion scale assesses general feelings of exhaustion (e.g., “Working all day is really a strain for me”), while the cynicism scale “assesses feeling of indifference or a distant attitude towards work; it represents dysfunctional coping with job strains” (e.g., “I have become less enthusiastic about my work”) [20] (p. 39). The professional efficacy subscale assesses an individual’s “feelings of effectiveness at work” and “encompasses both social and non-social aspects of occupational accomplishments” (e.g., “At my work, I feel confident that I am effective in getting things done”) [20] (p. 39). The reliabilities of the three subscales are good, with Cronbach alpha values of 0.83–0.90 for exhaustion, 0.74–0.80 for cynicism, and 0.70–0.83 for professional efficacy [20] (p. 41). As per scale instructions, the responses were summed within each subscale to give overall subscale scores, which were then compared to an international normative sample [20].

Additionally, we analysed the data using the burnout profile approach described by Leiter and Maslach [15]. Briefly, z-scores were calculated for each subscale to denote a cut-point for a “high” score and participants were then classified into one of five possible profiles: engaged (low exhaustion, low cynicism, high professional efficacy), ineffective (low professional efficacy), overextended (high exhaustion), disengaged (high cynicism), and burnout (high exhaustion, high cynicism).

The questionnaire also included questions to collect participants’ demographic data (i.e., age, self-identified gender, marital status, farming commodity, and province), level of satisfaction with support from spouse/romantic partner, family, friends, and industry (originally on a 5-point rating scale, where 1 = very dissatisfied and 5 = very satisfied, but dichotomized for analyses to: dissatisfied (very dissatisfied and dissatisfied) or satisfied (somewhat satisfied, satisfied, very satisfied)), level of financial stress (originally none, a little, some, or a lot, but dichotomized for analyses to yes (a little, some, a lot) or no (none)), and self-rated health (poor, fair, good, very good, or excellent). Two questions were asked regarding participants’ self-reported history of mental illness (“Are you currently on medication for mental illness (e.g., depression, anxiety, schizophrenia, bipolar disorder)?” and “Have you ever suffered from a mental illness in the past?”). The questionnaire was designed for a 15–20 min completion time.

### 2.3. Sample Size

A conservative estimate of 50% prevalence, an allowable error of 5%, and 95% confidence were used to estimate a required sample size for the national survey [21]. This resulted in a minimum required sample size of 385 farmers.

### 2.4. Statistical Analyses

Descriptive statistics (i.e., means, medians, standard deviations, interquartile ranges, and percentages) were used to describe the data. If there was one missing value within a MBI subscale, it was handled by person mean imputation; if more than one value was missing per MBI subscale, the observation was dropped from that analysis. If observations had missing data points from other questions, they were dropped from that analysis. Cronbach alpha values were calculated for each MBI subscale. T-tests were used to compare the sample population’s mean MBI subscale scores with the mean subscale scores from a large international normative sample with data from over 47,000 people [20] (p. 46). Fisher’s exact chi-square tests were used to test the association of gender with burnout profile. As a means of assessing the potential for response bias, we used an extrapolation method to compare early responders to late responders, where the latter were used as a proxy for non-responders [22]. Chi-square tests were used to determine whether significant differences in burnout subscale scores existed between early and late responders.

To explore factors associated with burnout, separate multivariable regression models were built with exhaustion, cynicism, and professional efficacy as the outcomes. Potential risk factors of interest were chosen based on previous research and a causal diagram. Correlations between variables were assessed using Spearman’s rank correlation coefficients, and were considered collinear when the correlation was >|0.8|. If collinear, the most biologically plausible variable was retained for further analysis. Continuous variables of interest were assessed graphically for linearity. Univariable regressions were conducted to screen variables for model inclusion; variables associated with the outcome at *p* < 0.20 were at least initially included in the multivariable models [21]. The multivariable models were built using a manual backwards approach with inclusion cut-off set at *p* < 0.05. Before being removed from the multivariable model, non-significant variables were assessed for confounding, which was defined as a > 20% change in the model coefficients upon removal [21]. The models for exhaustion and cynicism met the assumptions for linear regression modeling, whereas the model for professional efficacy did not. As such, professional efficacy was dichotomized into “high” or “low” based on the z-score described above, and a logistic regression model was created for this outcome. All pair-wise interactions of variables in the main effects model were considered. Based on previous research around mental health outcomes, age and gender were considered potential confounding variables and were forced in the final model regardless of statistical significance. For the linear models, model fit was tested using appropriate residual statistics and F-tests; for the logistic model of professional efficacy, model fit was assessed via appropriate residual statistics and the Hosmer–Lemeshow goodness of fit test [21]. Stata v.15 (StataCorp, College Station, TX, USA) was used for all statistical analyses.

## 3. Results

Responses to the MBI were received from 1075 participants; however, sample sizes per item vary, as noted, given that no questions were mandatory. The distribution of the study sample by farm type and province is presented and compared to 2016 national census data [23,24] in Table 1. A total of 53% (569/1075) of all participants were involved in more than one farming commodity. Approximately two-thirds of participants (671/1075) reported they grew crops, and of these, 500 (46.5% of all participants) grew crops in addition to producing at least one other commodity, and 171 (15.9%) reported only growing crops. The mean age of participants (*n* = 964) was 46.5 years (SD 13.3; IQR 35–57; range 19–88), which is lower than the mean age reported for farm operators in Canada (55.0 years) [25]. The self-reported gender breakdown of participants was 69.6% (670/962) male, 30.1% (290/962) female, and 0.2% (2/962) gender-queer. Given the small sample size and for reasons of anonymity, data in the gender-queer category are hereafter reported with the missing gender category. The observed gender breakdown amongst participants is similar to the sex breakdown reported for farm operators in Canada in 2016 (71.3% male and 28.7% female) [26]. Most participants were married (77.6%; 753/970) or in a committed relationship (10.1%; 98/970), while 9.2% (89/970) were single, 2% (19/970) were separated/divorced, 0.7% (7/970) were widowed, and 0.4% (4/970) described their relationship status as “other”. One-quarter (238/955) of participants reported that they previously experienced a mental illness, and 9% (88/969) reported currently taking medications for mental illness.

The results from the MBI are presented in Table 2. Participants’ mean exhaustion and cynicism scores (2.68 and 2.12, respectively) were significantly higher (*t* = 9.24, *p* < 0.00001 and *t* = 8.75, *p* < 0.00001, respectively) than reported norms from an international normative sample (2.26 and 1.74, respectively) [20]. Scores for professional efficacy were also significantly higher (*t* = 13.73, *p* < 0.00001) in our sample than population norms (4.85 vs. 4.34, respectively) [20].

**Figure 1:**
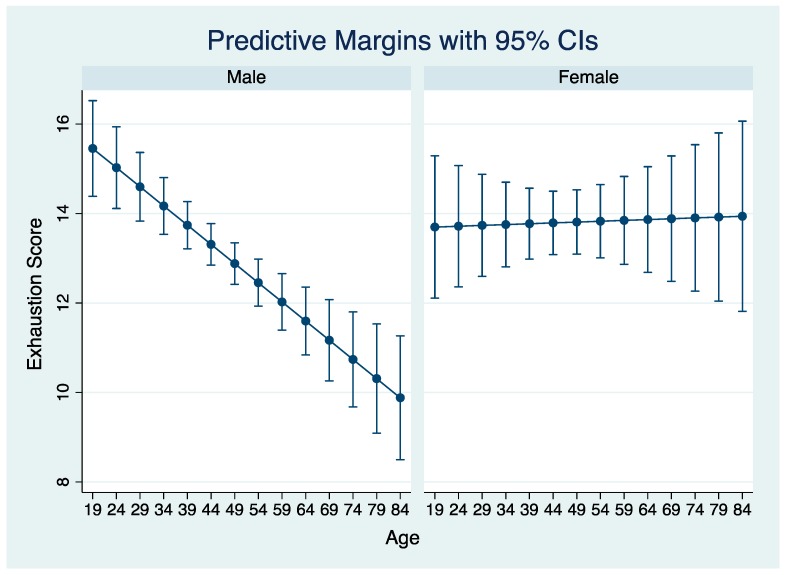
Predicted Maslach Burnout Inventory–General Survey exhaustion score by participant gender and age from a multivariable linear regression using data from 895 participating farmers in Canada (September 2015–February 2016).

The multivariable models of exhaustion, cynicism, and professional efficacy are depicted in Table 3. In the model of exhaustion, participants who reported having a previous mental illness scored significantly higher (1.39 points) on exhaustion compared to those without a history of mental illness. There was a positive relationship between exhaustion scores and cynicism scores, whereby each unit increase in cynicism was associated with a 0.6 point increase in exhaustion score. Participants in Alberta scored 1.73 points lower on exhaustion compared to farmers in Ontario. The model contained several significant interaction terms, which are interpreted in Figure 1 and Table 4A. The association of age with exhaustion score depended on gender. For males, increasing age was associated with decreasing exhaustion score, whereas scores for females remained relatively constant across all ages. Further, males tended to have higher exhaustion scores than females until approximately mid-thirties, after which females tended to have higher exhaustion score than males (Figure 1). The remaining interaction terms are interpreted in Table 4A. A financial stress increase was associated with an increase in exhaustion score (1.63 points when participants were satisfied with friend support), with a greater increase in exhaustion score when combined with dissatisfaction with support from friends (8.64 points). The association of satisfaction with industry support and exhaustion score depended on participant gender. Males who were dissatisfied with industry support had higher exhaustion scores than males or females who were satisfied with industry support. Finally, fair (vs. excellent) self-rated health was associated with increased exhaustion score (3.05 points), particularly when also being a dairy farmer (3.68 points).

**Figure 2:**
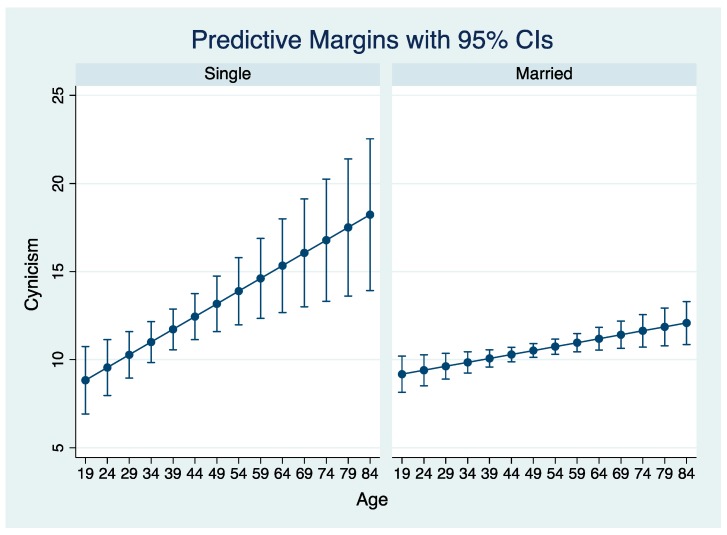
Predicted Maslach Burnout Inventory–General Survey cynicism score by participant gender and marital status from a multivariable linear regression using data from 876 participating farmers in Canada (September 2015–February 2016).

For the cynicism model, participants who were dissatisfied with the support provided by their industry scored 1.4 points higher on cynicism compared to participants who were satisfied with this support. There was a positive relationship between exhaustion score and cynicism, and a negative association between professional efficacy and cynicism scores. The model contained two significant interaction terms (Figure 2 and Table 4B). Cynicism scores increased with increasing age, and increases in scores were greater for single participants than participants who were married (Figure 2). Amongst males, being a dairy cattle farmer was associated with a lower exhaustion score (1.37 points) but being female and a dairy farmer was associated with a higher cynicism score (by 1.83 points).

Factors that were significantly associated with professional efficacy were all related to participants’ satisfaction with perceived supports. Compared to participants who were satisfied with these sources of support, dissatisfaction with support provided by a spouse/romantic partner, by a family member, and by the participants’ industry were all significantly associated with a lower professional efficacy score, approximately doubling the odds of being in the low professional efficacy category (Table 3). While age was found to have a statistically significant association with professional efficacy, the effect size was very small (*OR* = 0.99).

In terms of burnout profiles, 43.5% (425/978) of participants were classified as “engaged”, 23.1% (226/978) as “ineffective”, 19.0% (186/978) as “overextended”, 2.4% (23/978) as “disengaged”, and 12.1% (118/978) were classified in the “burnout” profile. There was a significant association between profile classification and gender, whereby females were more likely to be classified in the burnout profile than males (chi-square = 11.43; *p* = 0.001).

## 4. Discussion

This study is among only a few to investigate burnout in farmers, the first to separately explore risk factors for the three components of burnout amongst farmers, and the first to characterize farmers by MBI burnout profiles. Overall, our results highlight concerns with respect to burnout in farmers in Canada. Maslach, Jackson, and Leiter (2016) define burnout as “a crisis in one’s relationship with work” (p. 21) and is characterized by high exhaustion, high cynicism, and low professional efficacy [20]. We interpret our findings through the lens of the Conservation of Resources Theory, whereby psychological well-being is proposed as being related to a balance between work demands and the available resources [27]. Theoretically, when demands outweigh resources, or resources are otherwise insufficient in meeting demands, burnout occurs; burnout in turn, is associated with changes in behaviours and attitudes [28].

### 4.1. Exhaustion

The exhaustion subscale relates to feelings of being overextended, worn out, debilitated, and fatigued; it “captures the problem of lacking sufficient energy to make a useful and enduring contribution at work” [15] (p. 91). The mean participant exhaustion score observed here was significantly higher than that reported for an international normative sample [20].

A previous history of mental illness and fair (vs. excellent) self-rated health were both positively associated with exhaustion scores; it may be that these states contribute to feelings of fatigue and reduced ability to effectively engage at work. Numerous reasons could exist for the sparing effect of being from the province of Alberta vs. Ontario, including differences in agricultural industries or spurious findings; future research using stratified sampling by province could be helpful. As expected from known inter-correlations between the three MBI subscales [20], there was a positive association between cynicism score and exhaustion. The association of age with exhaustion depended on gender. Amongst males, exhaustion scores decreased with increasing age, but they remained largely constant amongst female participants. Males had higher exhaustion scores than females until ages approximately in the mid-thirties, after which females had higher scores. Potential reasons for observed gender differences are discussed in a separate section below.

Lee and Ashforth conducted a meta-analysis of correlates of the three dimensions of burnout as measured by the MBI–Human Services Survey (MBI–HSS), including emotional exhaustion [28], which is the subscale analogous with the exhaustion subscale of the MBI–GS. Emotional exhaustion was reported to be correlated with a number of work demands generally [28], including several that are common in farming: stressful events (e.g., disease outbreaks, extreme weather events, market instability), role conflict (e.g., conflict between various roles required in farming: animal care, planting/harvesting, business management, regulatory paperwork, sales/marketing, and between work and personal lives), workload, and work pressure. Thus, it is reasonable to consider that the demands associated with farming contribute to the high exhaustion scores observed here. Indeed, time pressure, long work days, and lack of free time were positively associated with burnout scores in Swiss farmers [19]. Financial stress was also associated with increased exhaustion scores in the present study, and this was greater when combined with dissatisfaction of support from friends and industry. Assistance with workload, work pressure, and financial stress could help counteract exhaustion, however, known issues with labour shortages, high employee turnover, and low employee retention [29] complicate the issue. Without addressing these systemic issues related to the agricultural workforce, it will likely be difficult to lessen farmers’ exhaustion in this way, particularly as farmers may feel increased pressure to expand and/or diversify to remain competitive (thus taking on additional financial stress), or seek off-farm employment to mitigate the economic risks associated with farming [30].

Emotional exhaustion is reportedly associated with negative behavioural and attitudinal outcomes, including job turnover intentions and decreased organizational commitment [28], hence, the high exhaustion results reported here may represent risks to the sustainability of agriculture. Addressing the agricultural workforce shortages and associated issues thus represents a crucial step in supporting farmers and Canadian agriculture; recommendations have been proposed elsewhere [29]. Resources reported to be negatively correlated with emotional exhaustion include social and work supports, peer cohesion, and autonomy [28]. Indeed, we found that dissatisfaction with both friend and industry supports were associated with increased exhaustion scores. Perhaps a strengthening of farmer supports provided by agricultural and commodity-specific organizations and via agricultural communities could help counteract some of the effects of farming demands and the resulting exhaustion.

### 4.2. Cynicism

The cynicism subscale “captures the difficulty in dealing with other people and activities” at work [15] (p. 91). The mean score observed here was significantly higher than an international normative sample [20].

The positive association between cynicism score and exhaustion score, and the negative association been cynicism and professional efficacy scores were expected based on the recognized inter-correlations between these three constructs [20]. Increased age was associated with increased cynicism scores, more so for participants who reported being single than being married. Perhaps the increase in cynicism with age is related to chronic stresses associated with farming in that cynicism is considered a coping mechanism whereby people withdraw from interpersonal stressors [20,28]. Marriage has been reported to be one of the most important sources of social support for farmers, where work and personal lives are so interdependent [31].

There was an interesting interaction with gender and dairy farming whereby this type of farming was associated with decreased cynicism scores if male, but increased cynicism score if female. This may be due to different gender roles (see below) but we cannot be conclusive based on these data; future investigation of burnout and its relation to gender roles and commodity groups would be informative. Finally, dissatisfaction with support from industry was positively associated with cynicism scores. Maslach and Leiter [20] report cynicism to be inherently associated with job environment, whereby poor quality of relationships at work and a lack of critical resources can lead to reduced job satisfaction and poor job performance. Industry support may serve as a critical resource to help offset farming demands. With respect to relationships at work, the line between work and personal lives is often blurred in farming; farmers often work where they live, work closely with family members, and farm-family conflicts are inherent in daily operations [32]. These realities may serve to increase cynicism scores in some farmers.

Depersonalization is a subscale in the MBI–HSS defined as, “an unfeeling and impersonal response towards recipients of one’s service, care, treatment, or instruction” [20] (p. 15) and is considered analogous to the MBI–GS’s cynicism subscale. Depersonalization has been found to be correlated with numerous demands, generally including several common to farming: role conflict, role stress, stressful events, and workload [28]. These farming demands may contribute to the cynicism results observed here. The high level of cynicism observed here is concerning; cynicism is reported to have negative impacts on job satisfaction and organizational commitment [28]. Resources correlated with depersonalization/cynicism are reported to include community bond, team cohesion, and skill utilization [28]. Hence, team building, creation of a climate that values all team members’ input [32], and division of labour based on individuals’ skills and strengths may be helpful.

### 4.3. Professional Efficacy

The professional efficacy subscale “captures the core self-evaluation people make regarding the value of their work and the quality of their contribution” [15] (p. 91). The overall professional efficacy score for participants was higher than international norms [20].

In our multivariable analyses of professional efficacy, satisfactions with support provided by spouse/romantic partner, family, and industry, were all positively associated with professional efficacy scores. It may be that feelings of being supported lends itself to positive self-perceptions of the value and quality of one’s work. Indeed, personal accomplishment (a subscale on the MBI–HSS that is analogous to professional efficacy and that “assesses feelings of competence and successful achievement in one’s work with people”) [20] (p. 15) is reportedly associated with work friends, participation, and team orientation [28]. While we did not investigate personal reasons behind feelings of professional efficacy, it has been reported that farmers find meaning and value in their roles as stewards of the land and caretakers of animals [33]. This view of farming as “a calling that provides purpose to their lives” [33] (p. 127) may also help explain our professional efficacy findings. Personal accomplishment/professional efficacy is positively associated with control coping [28], “suggesting that a problem-focused response and a positive self-appraisal may be mutually reinforcing” (p. 130). Other authors have reported that stress is less severe amongst farmers who feel confident in their ability to manage problems [31]. As control coping is a desired state, particularly in demanding roles like farming, a strengthening of personal resources and self-appraisal, work value, and social bonds could therefore be useful.

### 4.4. Profiles

Approximately 43% of participants were classified as ‘engaged’ meaning they had low scores for exhaustion and cynicism and high scores for professional efficacy. This is encouraging and represents a positive outcome for these farmers. Engagement is the ideal end of the burnout–engagement continuum and one that is “characterized by energy, involvement, and efficacy” [34] (p. 2). Given positive associations with productivity, engagement also represents the societal ideal in terms of the vital role that farmers serve in society and their contributions to national and global economies. The ineffective, overextended, and disengaged profiles (approximately 44% of our participants) may reflect transitional states towards burnout [15], and thus, represent cause for concern. Twelve percent of our participants met the classification for burnout, with high exhaustion, high cynicism, and low professional efficacy. Participants who identified as female were significantly more likely to be classified with the burnout profile than those who identified as males.

Although different instruments were used, our findings are strikingly in-line with Reissig et al. [19] who investigated burnout in farmers in Switzerland using the Copenhagen Burnout Inventory and reported 12% prevalence of burnout, with higher estimates among females (15%) than males (10.4%). Kallioniemi and colleagues also reported burnout symptoms being common amongst Finnish dairy farmers in a survey conducted in 2010 [8].

While there is a paucity of knowledge on burnout in farmers specifically, burnout is associated with numerous negative outcomes generally. A systematic review of prospective studies of burnout reported consequences to include physical (e.g., diabetes, cardiovascular disease, gastrointestinal issues, respiratory problems), psychological (e.g., insomnia, depression, psychological ill-health), and professional (e.g., job dissatisfaction, absenteeism, presenteeism) [16]. Further, burnout is also associated with lowered efficiency, reduced job performance, decreased commitment, and increased turnover, likely due to reduced coping capacity and decreased motivation [20]. It is thus reasonable to postulate that burnout could have implications for farm productivity, the retention of farmworkers and farmers, and for farm succession planning between generations; these in turn, may threaten agricultural sustainability.

### 4.5. Gender Differences in Burnout

Several gender differences in burnout were found in the present study. Male participants had higher exhaustion scores than females up until the approximate age of mid-thirties, after which, females had higher exhaustion scores than males, and more females had a burnout classification than males. Differences in gender roles amongst male and female farmers could help explain these findings. For example, females may experience added stress and exhaustion related to higher demands from role conflict, tending to multiple roles like working on-farm and off-farm, child-rearing, and management of the household [35]. Nevertheless, the complex relationship of sex and gender with health outcomes, influenced by work and life contexts and differences across the lifespan, should also be considered. Similar to our findings, in their meta-analysis, Purvanova and Muros (2010) found a difference in self-reported burnout for women, driven by differences in reported emotional exhaustion on the MBI. Those authors suggest when gender differences in self-reported burnout are detected, there is danger in assuming that burnout is a “female experience” [36]. It may be true that females in our study experienced more exhaustion (after mid-thirties) and burnout than males; however, it is also possible that differences in self-reported mental health status may be understood using a gendered social learning theory, whereby males may be less likely to express feelings of exhaustion because they learn to remain stoic, withdraw under stress, and conceal their emotions [37]. Hence, it is possible that gender differences observed here reflect differences in willingness and ability to disclose symptoms of distress amongst males and females [37], which in turn, could present the risk that male burnout may go unrecognized and thus, overlooked in interventions. The observation that males younger than middle-thirties had higher exhaustion scores than females of the same age is interesting and could reflect generational differences with respect to male willingness to disclose symptoms (whereby younger generations of males are more willing than older generations); however, we cannot conclude this based on these data alone. To elucidate the gendered findings in this study, further qualitative research is recommended.

While direct comparisons of risk factors are somewhat hindered as we investigated risk factors separately for the three components of burnout and Reissig et al. [19] investigated the overall burnout construct, our results support the findings of Reissig et al. in several respects. Reissig et al. reported that, for both male and female farmers in Switzerland, financial stress was positively associated with burnout score (exhaustion score in present study), while social support was negatively associated with burnout score (all three burnout subscales in the present study).

The importance of farmer supports is clear in both Reissig’s study and the present study, and may suggest important lines of intervention to help counteract the stressors inherent in farming and the associated negative impacts to mental health, including burnout. Increased opportunities for quality social interactions would likely be useful. For example, rural recreational activities are reported to result in improved quality of life, personal growth, enhancement of family solidarity, and community cohesion and pride (although the rural decline observed in many areas may threaten its sustainability) [38]. Farmer-to-farmer connections via industry events or social media may also help through the merits of social connection and peer-support. Where members of the community are trained in mental health literacy, such social interactions could pose the additional benefit of earlier recognition of signs shown by someone struggling with burnout or mental health more generally, and thus, also present a potential avenue for earlier intervention.

Our results support a rationale to develop interventions to reduce burnout and distress among Canadian farmers. Research on the impact of workplace conditions on one’s mental well-being has focussed on “identifying and, eventually, changing organizational conditions with the ultimate goal of improving well-being” [39] (p. 2). The stressors inherent in farming are unique and research based on monitoring organizational characteristics and the impact of implementing organizational change on the mental well-being of employees has limited generalizability to farmers at best. Indeed, from a meta-analysis, Maricuţoiu and colleagues recommended that burnout interventions be tailored for the population and focussed on all relevant aspects of burnout for the given population [40]. Hence, burnout interventions for farmers should be created such that they are tailored for farmers and the realities of agriculture, much as has been done for other occupations. For example, a meta-analysis of studies of interventions tailored for physician burnout reported that individual-focused interventions, such as mindfulness, stress management, small group discussions, and structural interventions can reduce burnout domain scores [41], although there is a need for more rigorous studies. A tailored approach for burnout interventions for farmers, ideally co-created using a participatory approach that includes farmers to ensure relevance and help with uptake, would be ideal for Canadian agriculture.

Our findings indicate that many Canadian farmers experienced exhaustion and cynicism about their work, while at the same time many reported high levels of professional efficacy and access to potentially helpful industry and social supports. Hence, some Canadian farmers are reacting to existing challenges, by engaging internal strengths and external supports that can be further leveraged.

Several limitations of this study should be noted, including the potential for selection bias. As a convenience sample was used, our sample does not proportionally represent the different farming commodities or provincial structure of agriculture in Canada, and our participants were slightly younger than the national average. Nevertheless, there were no significant differences (*p* > 0.05) in any of the burnout subscale scores (exhaustion, cynicism, or professional efficacy) when early responders were compared to late responders (data not shown) suggesting limited impact on burnout results. Regrettably, we did not offer a French translation of the survey which likely limited representation from French speaking farmers in the country. The use of formal sampling frames for random selection of participants and offering of the survey in both Canadian official languages would be useful in future. Further, we used only quantitative approaches here, whereas qualitative research approaches would be useful in better understanding the experience of burnout in farmers.

The classification of participants according to burnout profiles is relatively recent in the MBI’s long history, and it is suggested that use of this approach could be helpful in earlier recognition of individuals who may be at risk for developing burnout [15]. As burnout research amongst farmers grows, the adoption of this approach would be helpful in comparing populations and in strategizing practical outcomes.

## 5. Conclusions

Overall, our results demonstrate cause for concern with respect to farmer burnout in Canada. Exhaustion and cynicism scores amongst participants were higher than international norms, where 44% of participants were classified as ineffective, overextended, or disengaged (intermediate profiles on the engagement–burnout continuum), and 12% of participants were classified as having burnout. This is concerning not only for the affected individuals and their families, but also given potential associated risks to Canadian agriculture via lowered productivity. These results serve as a call to action for increased farmer supports to decrease work demands and increase resources, particularly through addressing systemic issues related to workload and via positive family, friend, community, and industry support and engagement.

## Figures and Tables

**Table 1:** Distribution of farm type and province in the study sample compared to 2016 Canadian census data [23,24].

Characteristic	Study Sample	2016 Census
**Farm Type**	***N* = 1075 # (%)**	***N* = 193,492 # (%)**
Dairy cattle	387 (36.0)	10,525 (5.4)
Beef cattle	233 (21.7)	36,013 (18.6)
Pigs	118 (10.9)	3305 (1.7)
Broiler chicken	111 (10.3)	2175 (1.1)
Layer chicken	89 (8.3)	2008 (1.0)
Turkeys	35 (3.3)	294 (0.15)
Sheep (meat and dairy combined)	108 (10.0)	2189 (1.1)
Goat (meat and dairy combined)	35 (3.2)	867 (0.44)
Other animal	42 ^a^ (3.9)	18,143 ^b^ (9.4)
Crops	671 (62.4)	90,011 (46.5)
Horticulture	49 (4.6)	20,547 (10.6)
Other farming ^c^	19 (1.8)	7415 (3.8)
**Number of farmers by province**	***N* = 1075 # (%)**	***N* = 271,935 # (%)**
Ontario	774 (72.0)	70,470 (25.9)
Alberta	97 (9.0)	41,995 (15.4)
British Columbia	58 (5.4)	4630 (1.7)
Manitoba	51 (4.7)	3005 (1.1)
Saskatchewan	49 (4.6)	500 (0.2)
New Brunswick	16 (1.5)	20,140 (7.4)
Nova Scotia	12 (1.1)	45,350 (16.7)
Quebec	10 (0.9)	57,605 (21.1)
Newfoundland and Labrador	5 (0.5)	26,430 (9.7)
Prince Edward Island	3 (0.3)	1810 (0.7)

^a^ Other poultry, rabbits, veal, bison, equine, fish, and game birds. ^b^ Other poultry, equine, fur-bearing, animal combination, and other miscellaneous animal production. ^c^ Apiculture and maple syrup.

**Table 2:** Maslach Burnout Inventory–General Services mean and median subscale scores (possible range: 0–6) amongst participating farmers in Canada (September 2015–February 2016).

	*n*	Mean (SD ^a^)	Median (IQR ^b^)
**Exhaustion subscale (Cronbach-alpha: 0.93)**			
Total	1075	2.68 (1.63)	2.6 (1.2–4.0)
Male	670	2.57 (1.63)	2.4 (1.2–3.8)
Female	290	2.91 (1.63)	2.8 (1.4–4.2)
Gender not reported ^c^	115	2.72 (1.58)	2.6 (1.4–4.2)
**Cynicism subscale (Cronbach-alpha: 0.84)**			
Total	1005	2.12 (1.47)	1.8 (1.0–3.2)
Male	647	2.06 (1.42)	1.8 (1.0–3.0)
Female	287	2.27 (1.50)	2.0 (1.2–3.4)
Gender not reported ^c^	71	2.05 (1.75)	1.4 (0.8–3)
**Professional efficacy subscale (Cronbach-alpha: 0.82)**			
Total	1008	4.85 (1.01)	5.2 (4.3–5.7)
Male	651	4.88 (0.98)	5.0 (4.3–5.7)
Female	288	4.75 (1.08)	5.2 (4.0–5.8)
Gender not reported ^c^	69	4.87 (1.10)	5.3 (4.0–5.8)

^a^ Standard Deviation. ^b^ Interquartile Range. ^c^ includes 2 participants who identified as gender-queer; their results have not been reported separately for reasons related to anonymity and small group size.

**Table 3:** Multivariable linear regression models of the exhaustion, cynicism, and professional efficacy subscales of the Maslach Burnout Inventory–General Survey for participating farmers in Canada (September 2015–February 2016).

**Exhaustion model (*n* = 895)**	**Beta coefficient**	**95% CI**	***p*-value**
**Main effects:**			
Age ^a^	−0.08	−0.12, −0.05	<0.001
Gender ^a,b^ (Referent: male)	−2.81	−5.86, 0.23	0.070
Financial stress ^b^ (Referent: no stress)	1.63	0.45, 2.81	0.007
Dissatisfaction with friend support ^b^ (Referent: satisfaction)	−6.41	−11.66, −1.16	0.017
Dissatisfaction with industry support ^b^ (Referent: satisfaction)	2.63	1.38, 3.87	<0.001
Previous self-reported history of mental illness (Referent: no illness)	1.39	0.47, 2.31	0.003
Dairy cattle farmer ^b^ (Referent: not dairy farmer)	−0.01	−2.25, 2.22	0.992
Fair self-rated health ^b^ (Referent: excellent)	3.05	1.22, 4.88	0.001
Alberta (Referent: Ontario)	−1.73	−3.08, −0.39	0.012
Cynicism score	0.60	0.55, 0.66	<0.001
**Interaction terms:**			
Age x Gender interaction ^a^	0.09	0.03, 0.15	0.005
Financial stress x Satisfaction with friend support interaction ^b^	8.64	3.21, 14.08	0.002
Gender x Satisfaction with industry support interaction	−2.62	−4.59, −0.65	0.009
Self-rated health (fair) x Dairy cattle farmer interaction	3.68	0.55, 6.80	0.021
**Cynicism model (*n* = 876)**			
**Main effects:**			
Age ^c^	0.14	0.06, 0.23	0.001
Gender ^b^ (Referent: male)	−0.77	−1.68, 0.14	0.095
Dairy cattle farmer ^b^ (Referent: not dairy farmer)	−1.37	−2.22, −0.52	0.002
Married ^c^ (Referent: single)	2.22	−1.50, 5.95	0.241
Dissatisfaction with support from industry (Referent: satisfaction)	1.40	0.50, 2.29	0.002
Exhaustion score	0.74	0.55, 0.94	<0.001
Professional efficacy score	−0.17	−0.29, −0.06	0.002
**Interaction terms:**			
Female gender x Dairy farmer interaction	1.83	0.20, 3.46	0.027
Age x Married interaction	−0.10	−0.19, −0.01	0.034
**Professional efficacy model ^d^ (*n* = 911)**	**Odds ratio**	**95% CI**	***p*-value**
Age	0.99	0.98, 1.00	0.023
Gender (Referent: male)	1.10	0.82, 1.48	0.538
Dissatisfaction with support from spouse/romantic partner (Referent: satisfaction)	0.54	0.31, 0.95	0.033
Dissatisfaction with support from family (Referent: satisfaction)	0.51	0.32, 0.80	0.003
Dissatisfaction with support from industry (Referent: satisfaction)	0.56	0.40, 0.79	0.001

^a^ Part of interaction term; to interpret refer to Figure 1. ^b^ Part of interaction term; to interpret refer to contrasts in Table 4. ^c^ Part of interaction term; to interpret refer to Figure 2. ^d^ Low-professional efficacy is the referent category.

**Table 4:** Contrasts derived from multivariable linear regression models for (A) exhaustion (to interpret effects of gender, financial stress, satisfaction with friend support, satisfaction with industry support, self-rated health, and being a dairy farmer) and (B) cynicism subscales (to interpret effects of gender and being a dairy farmer) of the Maslach Burnout Inventory–General Survey for participating farmers in Canada (September 2015–February 2016).

Interaction Term	Contrast	Beta Coefficient	95% CI	*p*-Value
**A. Exhaustion Model**				
Financial stress x Satisfaction with friend support (Referent: no financial stress & satisfied with friend support)	Financial stress & satisfied with friend support	1.63	0.45, 2.81	0.007
	No financial stress & dissatisfied with friend support	−6.41	−11.66, −1.15	0.017
	Financial stress & dissatisfied with friend support	8.64	3.21, 14.08	0.002
Gender x Satisfaction with industry support (Referent: male gender & satisfied with industry support)	Male gender & dissatisfied with industry support	2.63	1.38, 3.87	<0.001
	Female gender & satisfied with industry support	−2.81	−5.86, 0.23	0.070
	Female gender & dissatisfied with industry support	−2.61	−4.59, −0.65	0.009
Fair self-rated health x dairy farmer (Referent: excellent self-rated health & non-dairy farmer)	Excellent self-rated health & dairy farmer	−0.01	−2.25, 2.22	0.992
	Fair self-rated health & non-dairy farmer	3.05	1.22, 4.88	0.001
	Fair self-rated health & dairy farmer	3.68	0.55, 6.80	0.021
**B. Cynicism model**				
Gender x dairy farmer (Referent: male gender & non-dairy farmer)	Female gender & non-dairy farmer	−0.77	−1.68, 0.14	0.095
	Male gender & dairy farmer	−1.37	−2.22, −0.52	0.002
	Female gender & dairy farmer	1.83	0.20, 3.46	0.027
